# Economic Evaluation of Healthcare Resource Utilization and Costs for Newly Diagnosed Dementia-Related Psychosis

**DOI:** 10.3390/geriatrics7020029

**Published:** 2022-03-08

**Authors:** Nazia Rashid, James B. Wetmore, Muna Irfan, Victor Abler

**Affiliations:** 1 Acadia Pharmaceuticals Inc., San Diego, CA 92130, USA; vabler@acadia-pharm.com; 2 Division of Nephrology, Hennepin County Medical Center, Minneapolis, MN 55415, USA; james.wetmore@hcmed.org; 3 Chronic Disease Research Group, Hennepin Healthcare Research Institute, Minneapolis, MN 55404, USA; 4 Veterans Affairs Medical Center, University of Minnesota, Minneapolis, MN 55417, USA; muna.irfan@hcmed.org

**Keywords:** dementia, hallucinations, delusions, psychosis, economic burden, Medicare

## Abstract

This retrospective cohort study described changes in all-cause healthcare resource utilization (HCRU) and associated costs in dementia patients newly diagnosed with psychosis. Dementia and incident psychosis were identified using diagnostic and pharmacy claims using a Medicare 20% random sample dataset. All-cause HCRU and unweighted and weighted (by person-years of follow-up) HCRU-associated costs were evaluated in the year prior to and the 4 years following diagnosis of psychosis. In 49,509 dementia patients with psychosis, physician visits per patient per year increased from a mean of 26.7 (standard deviation (SD) 20.0) prior to psychosis to 38.4 (SD 41.9) post-psychosis diagnosis. The number of inpatient stay claims increased from 1.0 (SD 1.4) to 1.7 (SD 5.8). Mean unweighted costs for inpatient stays and home healthcare/hospice during 2008–2016 were USD 9989 and USD 3279 prior to a diagnosis of psychosis but increased to USD 25,982 and USD 9901 (weighted: USD 11,779 and USD 6709), respectively, in the year after a psychosis diagnosis. This pattern of a sharp increase in mean costs was also observed in costs adjusted to 2015 USD, and in both unweighted and weighted total and psychosis-related costs. These results indicate the importance of identifying newly diagnosed psychosis in dementia patients as well as the pressing need for management strategies and treatments that can reduce HCRU and costs.

## 1. Introduction

The natural course of dementia involves progressive neurodegeneration, a process accompanied by new and worsening symptoms, heightened caregiver burden, and increased utilization of medical and long-term care (LTC) resources [1]. Neuropsychiatric symptoms (NPS) are common in dementia, particularly in advanced stages of disease, and often contribute severely to patient and caregiver burden [2,3]. Healthcare resource utilization (HCRU) and associated costs are higher in patients with dementia-related NPS compared with dementia alone [4] and increase as NPS severity worsens [3,4,5]. In one report, patients who had dementia with NPS incurred nearly USD 10,000 more in total healthcare costs per patient per year, relative to patients who had dementia without NPS, with the costs of inpatient stays constituting more than half of this additional cost burden [4]. 

Certain types of NPS are more likely than others to require HCRU. For example, dementia-related hallucinations and delusions may generate more acute and urgent needs, such as outpatient or emergency department (ED) visits, inpatient stays/hospitalizations, or admission to LTC facilities [2]. In contrast, apathy or depression may lead only to outpatient physician visits. Relative to patients with dementia alone, patients with dementia-related psychosis experience worse outcomes, including more rapid cognitive and functional decline [6,7], and an increased risk of institutionalization and death [8,9], both of which may also translate to increased HCRU and costs. Specifically, patients with dementia who experience hallucinations and delusions are at a 1.6 times higher risk of institutionalization relative to patients without dementia-related psychosis [9]. Institutionalization is a costly transition for patients with dementia-related psychosis and their caregivers [10]. Patients who suffer from dementia-related psychosis are also more prone to falls than those with dementia alone, which can lead to acute hospitalizations and an associated increase in costs [11,12]. No therapies are approved to treat hallucinations and delusions in patients with dementia in the United States [13], and existing off-label treatments, including antipsychotics, are associated with uncertain benefits and considerable safety concerns [14,15,16]. Current American Psychiatric Association guidelines caution against the use of such agents in these patients except when symptoms are severe, dangerous, and/or cause significant distress to the patient [14,15,16].

A number of studies have assessed HCRU and costs associated with dementia and dementia-related NPS [1,2,4,17,18], but HCRU and costs associated with the presence of dementia-related hallucinations and delusions have rarely been evaluated separate from other NPS [12]. To better understand whether the economic burden increases following occurrence of dementia-related psychosis, we conducted a Medicare claims-based descriptive analysis that evaluated all-cause and psychosis-related HCRU and associated costs during the year before diagnosis and during follow-up in dementia patients newly diagnosed with psychosis.

## 2. Materials and Methods

### 2.1. Data Source and Study Design

This retrospective cohort study used the 20% Medicare random sample of beneficiaries enrolled at any time between 1 January 2008 and 31 December 2016. Methods used here to identify patients with dementia and dementia-related psychosis were described in a previous report analyzing the risk of death and use of long-term care associated with dementia-related psychosis in the same patient population [19]. All data were compliant with the Human Subjects Research Committee of the Hennepin County Medical Center/Hennepin Healthcare System, Inc. This study included de-identified patient-level data; institutional review board approval and patient consent were not required.

### 2.2. Patient Population

Medicare beneficiaries ≥ 40 years old with Medicare fee-for-service coverage (Parts A, B, and D) for at least 1 year before the dementia index date met criteria for dementia-related psychosis. Patients with dementia were identified based on 1 of the following sets of criteria: ≥2 dementia diagnosis codes (determined according to International Classification of Diseases, Ninth and Tenth Revisions (ICD-9 or ICD-10), Clinical Modifications codes; Supplemental Table S1) separated by ≥30 days (but less than 3 years apart), with the later date considered the dementia index date, or 1 dementia diagnosis code plus ≥ 1 dementia prescription (including donepezil hydrochloride, galantamine hydrobromide, rivastigmine, tacrine hydrochloride, memantine HCL) within 1 year before or after the diagnosis claim date, where the later date was considered the dementia index date. If a patient met both criteria for dementia, the earlier index date was used. Patients carrying a pre-existing diagnosis of chronic psychiatric disease, history of seizures, history of chronic alcohol-induced dementia or drug-induced persisting dementia, or history of stroke less than 6 months before the dementia index date were excluded from analyses (Supplemental Table S2). 

Patients with newly diagnosed psychosis were identified from patients identified with dementia. Patients with incident dementia-related psychosis were identified based on 1 of the following criteria: (1) ≥2 diagnosis codes for psychosis (Supplemental Table S3) after the dementia diagnosis ≥ 7 days (but less than 3 years) apart (to minimize the possibility of capturing an acute, highly transient psychotic episode), where the later date was designated the psychosis index date; (2) ≥2 prescriptions for dementia-related psychosis treatment (aripiprazole, asenapine, clozapine, divalproex, haloperidol, iloperidone, lurasidone, olanzapine, paliperidone, quetiapine, risperidone, sulpiride, or ziprasidone) after the dementia diagnosis and ≥180 days apart (suggesting chronic use), where the second prescription date was designated the psychosis index date, or (3) 1 diagnosis code for psychosis plus ≥ 1 prescription for dementia-related psychosis treatment within 1 year before or after the psychosis diagnosis, where the later date was designated the psychosis index date. To exclude patients with pre-existing psychosis unrelated to dementia, patients who had evidence of psychosis, behavioral abnormalities, or antipsychotic drug use before the dementia date were excluded from the analysis.

### 2.3. Outcomes

Baseline demographics and comorbid conditions were identified for patients with dementia only and incident dementia-related psychosis. Comorbid conditions were defined using qualifying diagnosis codes from ≥1 inpatient, skilled nursing facility (SNF), or home healthcare/hospice (HH/HS) claim or ≥2 outpatient ED/observation stays, physician visits, or durable medical equipment (DME) claims on different days during the baseline period. Comorbid conditions were identified according to the Charlson Comorbidity Index [20].

We report two major outcomes: HCRU and costs. HCRU is assessed in the form of claims generated for services, as described more fully below. For Medicare costs, we assessed Medicare allowable costs, which include costs paid by Medicare as well as by patients. Allowable costs, which can be considered as representing the perspective of both the entitlement program payer (Medicare) and the patient, best represent total costs. The HCRU and Medicare allowable cost outcomes are reported as both all-cause and cause-specific for dementia-related psychosis, the latter of which represents the specific economic burden on healthcare systems and payers associated with the emergence of dementia-related psychosis. All-cause and psychosis-related HCRU and associated costs were assessed for the 12 months before the dementia-related psychosis index date (baseline period) and during the follow-up period. The follow-up period was categorized into yearly increments. The follow-up period began on the index date and continued until death, loss of Medicare Parts A, B, or D eligibility, or end of the study period (31 December 2016), whichever came first. Data for the first 4 years of post-index follow-up are reported here. No discount rates were used to interpret changes in costs during follow-up.

For both all-cause and psychosis-related analyses, HCRU was evaluated for the following: inpatient stays (hospitalizations), ED/observation stays, nonemergency unscheduled outpatient visits, SNF, HH/HS, physician visits, prescriptions for DME, and prescription drug fills. The mean number of claims per patient per year and annualized costs were evaluated overall and for each setting. All-cause Medicare-covered prescription drug claims and costs were estimated using Medicare Part D claims. Prescription drug claims were counted according to the actual number of claims. 

Initially, costs were annualized and evaluated as unweighted means, which are commonly reported. Due to person-years of follow-up time varying by patient (e.g., one patient may be fully observable for an entire year within a given year of data, while another may be fully observable for only some portion of the year), we accounted for variation of follow-up time using weighted means (i.e., using person-years as weight) when determining annualized allowable costs. Both unweighted and weighted costs were adjusted for inflation, expressed as 2015 USD, and measured according to the Medicare allowable costs. LTC stays were identified from a Centers for Medicare and Medicaid Services linkage database with data on LTC Minimum Data Set and Medicare claims. LTC-associated costs were estimated by calculating the length of LTC stay (in days) multiplied by 2015 room rates, using an average of semiprivate and private rates [21]. Patients living in the same state shared the same daily LTC costs. LTC costs were annualized by dividing total cost by the total follow-up time in months multiplied by 12. 

For psychosis-related HCRU and cost, claims with psychosis diagnosis codes or prescription drug claims with antipsychotic drugs were examined. Any identified psychosis-related HCRU and costs in the pre-index period refer to those associated with the first psychosis diagnosis code or prescription drug fill for psychosis treatment before the psychosis diagnosis was confirmed, which determined the psychosis index date. Psychosis-related LTC costs were not calculated/reported due to unavailability.

### 2.4. Statistical Analysis

HCRU and cost results were calculated using descriptive statistics using SAS version 9.4 (SAS Institute, Cary, NC, USA). Annualized HCRU was calculated using the total number of claims divided by the total follow-up time (in months) multiplied by 12. The HCRU associated costs were evaluated as unweighted and weighted means. 

## 3. Results

### 3.1. Patient Demographics and Characteristics

During 2008–2016, 206,899 patients with dementia only and 49,509 patients with both dementia and incident dementia-related psychosis met all inclusion criteria and were included in the analysis (Supplemental Figure S1). Baseline patient characteristics have been published previously [19]. At the dementia index date, approximately two-thirds (68.0%) of patients with dementia-related psychosis were 76–90 years old, 71.0% were female, 85.7% were white, and 64.6% had ≤1 comorbid condition [19]. Common comorbid conditions included diabetes without chronic complications, chronic pulmonary disease, peripheral vascular disease, and congestive heart failure.

### 3.2. All-Cause HCRU and Associated Costs for Patients with Dementia-Related Psychosis

For all settings, the unweighted mean annualized number of all-cause HCRU claims per patient per year increased from the year before dementia-related psychosis diagnosis (baseline period) to the years after diagnosis (follow-up period; Table 1). The first year post index was characterized by increased mean all-cause HCRU relative to the baseline period and by the highest mean HCRU of all years analyzed. The number of inpatient stay claims overall increased from 1.0 ± 1.4 in the year prior to psychosis to 1.7 ± 5.8 in the year post-psychosis diagnosis. The number of physician visits per patient per year increased from a mean of 26.7 ± 20.0 prior to psychosis to 38.4 ± 41.9 post-psychosis diagnosis. In most settings, HCRU gradually declined in subsequent years, eventually settling at rates similar to or lower than baseline by the fourth year post index. However, at 4 years post index, HH/HS claims and prescription drug fills remained at a mean of 1.4 claims and 14.8 fills higher than the baseline period prior to diagnosis, respectively. Throughout the follow-up period, the post baseline mean number of all-cause prescription drug fills remained ≥14.0 fills per patient per year above baseline. Annualized all-cause weighted HCRU claims per patient per year for patients with dementia-related psychosis are shown in Supplemental Table S4. 

**Figure 1:**
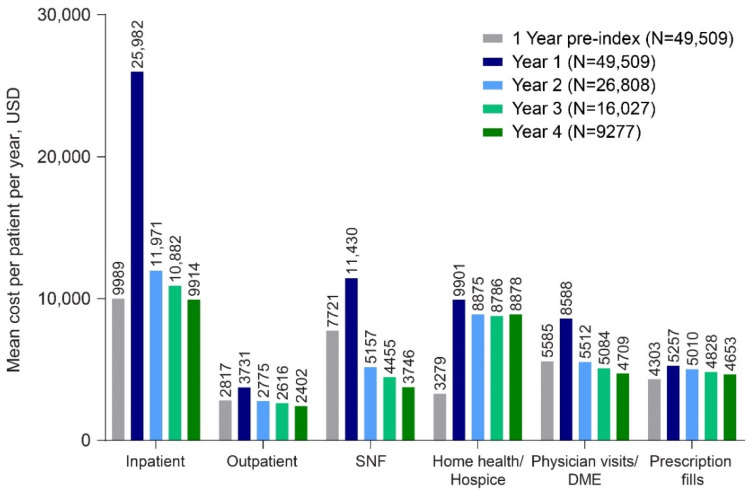
Annualized unweighted all-cause costs per patient per year by type of Medicare setting adjusted to 2015 USD. LTC and Medicare costs may not sum to total row costs due to rounding. Abbreviations: DME = durable medical equipment; LTC = long-term care; SNF = skilled nursing facility.

Unweighted and weighted mean all-cause HCRU-associated costs adjusted to 2015 USD also showed a substantial increase in the first year post index (Figure 1, Supplemental Table S5). At all of the time points, inpatient, SNF, and HH/HS costs accounted for the largest proportion of increased Medicare costs. The largest increases in mean cost per patient per year were observed for inpatient stays, with unweighted mean costs increasing from USD 9989 during the baseline period to USD 25,982 during the first follow-up year, and for HH/HS, with unweighted mean costs increasing from USD 3279 during the baseline period to USD 9901 during the first follow-up year (Figure 1). Weighted mean costs per patient per year in the first year following a psychosis diagnosis for inpatient stays and HH/HS also showed the largest increases (USD 11,779, inpatient stays; USD 6709, HH/HS) (Supplemental Table S5). Across all categories, mean costs had large SDs, indicating that data were not normally distributed (Supplemental Table S5). 

**Figure 2:**
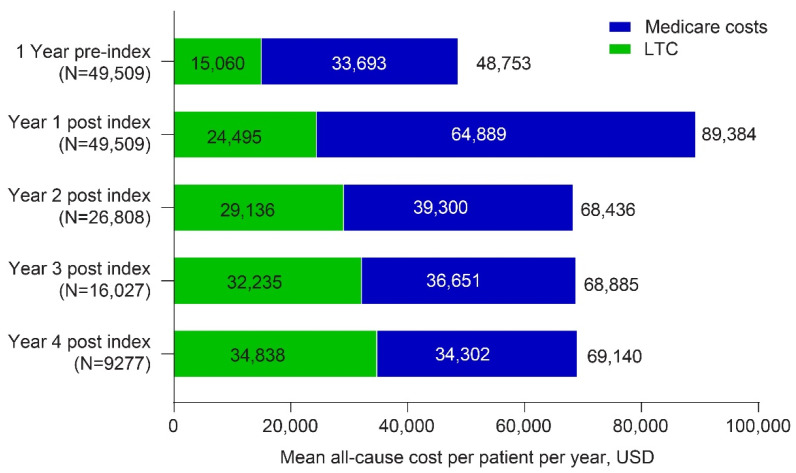
Annualized unweighted all-cause costs per patient per year overall in Medicare and LTC in 2015 USD. Abbreviation: LTC = long-term care.

Unweighted and weighted mean all-cause LTC costs, which were calculated separately from Medicare costs, also had the greatest increase from pre-index in the year following the start of dementia-related psychosis (Figure 2) but then increased steadily over time. Unweighted mean LTC costs increased from USD 15,060 to USD 24,495 in the first year following a psychosis diagnosis. Weighted mean LTC costs per patient per year were USD 26,270 in the first year following a psychosis diagnosis (Supplemental Table S5). When both Medicare and LTC costs were evaluated together, unweighted mean total costs increased from USD 48,753 during the baseline period to USD 89,384 in the first year post psychosis (weighted mean costs per patient per year were USD 67,704 in the first year post psychosis). Mean total costs decreased from year 1 in subsequent years but remained above baseline costs through 4 years post index (Figure 2, Supplemental Table S5).

### 3.3. Psychosis-Related HCRU and Associated Costs

**Figure 3:**
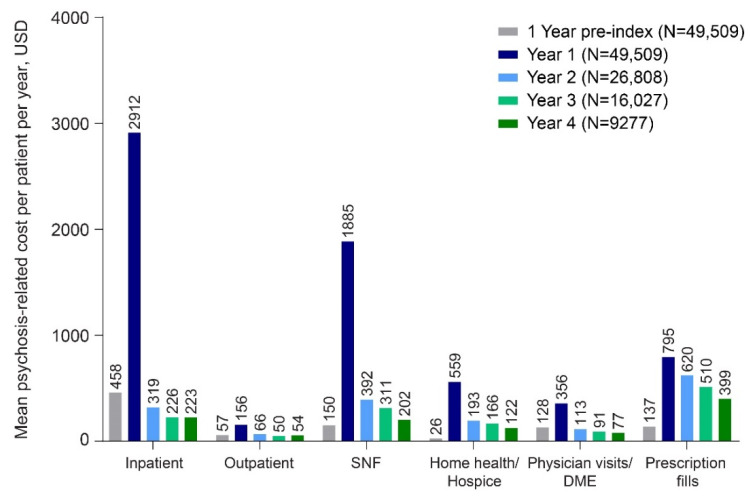
Annualized unweighted psychosis-related costs per patient per year by type of Medicare Setting in 2015 USD. LTC and Medicare costs may not sum to total row costs due to rounding. Abbreviations: DME = durable medical equipment; LTC = long-term care; SNF = skilled nursing facility.

Patterns for psychosis-related HCRU and costs over time were similar to all-cause HCRU and costs, with increases in number of claims (Table 2) and in both unweighted and weighted mean cost values but abnormally distributed data in most settings (Figure 3, Supplemental Table S6). The most frequently used psychosis-related resources were prescription drug fills and physician visits, for which the unweighted mean number of claims increased 3.6-fold (2.11 ± 3.37 to 7.66 ± 9.21) and 2.8-fold (0.77 ± 0.95 to 2.17 ± 9.19) between the baseline period and the first year of follow-up. The annual number of mean ED encounters/observation stays were the exception to this pattern, as these dropped from 0.04 ± 0.22 during the baseline period to 0.03 ± 0.49 in the first year after psychosis diagnosis (Table 2), then remained at 0.00–0.01 thereafter. Psychosis-related HCRU claims dropped to near or below baseline values by year 2 for most settings, but prescription drug fills remained elevated throughout the study period. Annualized psychosis-related weighted HCRU claims for patients with dementia-related psychosis are shown, analogously, in Supplemental Table S7.

As with mean all-cause allowable costs, in most settings, the weighted and unweighted mean psychosis-related costs in 2015 USD increased substantially from baseline and were highest during year 1, then decreased during subsequent years to near baseline levels (Figure 3, Supplemental Table S6). Unweighted mean total psychosis-related Medicare costs were USD 955 in the year pre-index, increased to USD 6663 (weighted mean costs, USD 3824) in the year post index, and remained above baseline in subsequent years, USD 1703 in year 2 (weighted mean costs, USD 1565), USD 1354 in year 3 (weighted mean costs, USD 1280), and USD 1077 in year 4 (weighted mean costs, USD 1045). Inpatient and SNF costs accounted for the largest proportions of psychosis-related total Medicare costs at any time, with the highest values of USD 2912 and USD 1885 (unweighted), respectively, during year 1 (Figure 3). Mean HH/HS costs also showed a large increase from the baseline period to year 1 (USD 26 to USD 559, unweighted), and mean costs in later years (USD 122 to USD 93, unweighted) remained above baseline levels.

## 4. Discussion

In this large-scale analysis of the Medicare beneficiaries, we found compelling increases in mean HCRU and associated costs in the year following the appearance of newly diagnosed psychosis in patients with dementia. This descriptive analysis revealed an increased economic burden across all the different setting types researched in this study, including inpatient, outpatient, SNF, HH/HS, physician visits, prescription fills, and use of LTC. In the year following a new presentation of psychosis, the mean annualized per patient per year all-cause Medicare and LTC costs in 2015 USD for dementia-related psychosis patients increased by about USD 40,000, reaching nearly USD 90,000 during the year after the psychosis diagnosis. 

Costs driven by inpatient stays increased from approximately USD 10,000 to USD 26,000 in the post-index year, suggesting that the initial manifestation of psychosis symptoms for patients with dementia is associated with disruptive and costly hospitalizations. Further, HH/HS costs increased from approximately USD 3000 to USD 10,000 in the first year post index and remained more than USD 5000 higher than baseline for at least 4 years post index. Increases in costs associated with HH/HS and prescription drug fills remained elevated for at least 4 years post index, suggesting that the development of psychosis may trigger a new trajectory for patients with dementia that is associated with persistent increased healthcare needs. 

The greatest proportional increases in psychosis-related costs were for inpatient stays, SNF, and prescription drug costs, with annualized per patient costs of approximately USD 2900, USD 1900, and USD 800, respectively, in the first year following psychosis diagnosis. While psychosis-related HCRU and costs accounted for only a portion of all-cause HCRU and costs in our analysis, it is possible that not all psychosis-related costs are adequately captured by analyzing claims data, particularly if the treating physician identifies dementia rather than the psychotic symptoms associated with dementia on the claim [1]. Due to be there are not being any standardized diagnosis codes for dementia-related psychosis and there is currently no approved treatment for hallucinations and delusions associated with dementia-related psychosis [13,14], treatments prescribed to address these symptoms may not be coded as such. 

The highest Medicare costs were observed during this first year following a new psychosis diagnosis; however, LTC costs continued to increase over time. This latter finding would be expected, as the risk of entering an LTC facility increases as the time since a dementia-related psychosis diagnosis increases [9,22]. The lasting increase in LTC costs observed here further supports the notion that symptoms of psychosis can lead to long-term increases in healthcare costs for patients with dementia.

The increase in all-cause claims and weighted and unweighted costs immediately following the index date might indicate that a diagnosis of dementia-related psychosis is often not made until symptoms become severe enough to warrant specific medical attention. Although dementia-related psychosis can be diagnosed at all stages of dementia [8], the diagnosis is often made in advanced stages [23], dementia-related psychosis often has an insidious onset and is progressive such that patients and caregivers might ignore symptoms until they become severe enough to affect quality of life [24]. If symptoms are intermittent or co-occur with other NPS, early signs of psychosis may be overlooked or misdiagnosed as depression or anxiety [25,26]. The lack of approved treatments for dementia-related psychosis may also slow clinicians in identifying psychosis. 

Though beyond the scope of the current analysis, future studies could examine how costs associated with dementia-related psychosis are related to symptom severity. Our findings are consistent with existing reports of increased costs associated with a dementia diagnosis and with the presence or worsening of NPS in patients with dementia [1,4,5,12,17]. One study reported that Medicare expenditures in the 2 years following a diagnosis of dementia increased 2- to 3-fold in the year after the diagnosis (relative to pre-diagnosis and patients without dementia) [1]. In two other studies, annual Medicare HCRU-related costs reported among patients with Parkinson’s disease psychosis, and among patients with behavioral or psychological symptoms of dementia (including hallucinations, delusions, and agitation) aligned with the cost estimates reported here [2,12]. These studies point to psychosis as the main factor that increases healthcare costs, rather than dementia or other behavioral symptoms such as agitation. In a study of patients with probable Alzheimer’s disease, patients with a high Neuropsychiatric Inventory (NPI) score (i.e., more severe NPS) had formal and total direct costs USD 3000 to USD 6000 and USD 10,500 to USD 16,000 higher than patients with a low NPI score, and a one-point increase in NPI score was associated with an annual increase in total direct costs of USD 250 to USD 400 [5]. Our findings are also consistent with a recent report that patients with dementia-related psychosis were more likely to have diagnoses for behavioral health conditions and experience clinical events, and, consistently, had higher mean all-cause and dementia-related HCRU, relative to those with dementia only [27]. 

## 5. Limitations

Our study has several important limitations. Results may not generalize to individuals outside of the United States. An assessment of claims data cannot account for indirect and nonmedical costs, which are also known to be high in patients with dementia-related NPS [1]. While the study methodology used a specific approach designed to focus on patients in whom psychosis was likely related to dementia, our approach to identifying dementia-related psychosis using claims data has not been validated, and some patients may have been misclassified. However, this limitation is offset by the large sample size and use of a nationally representative administrative database. This study was a descriptive analysis, further economic studies need to be conducted evaluating factors associated with high or low costs in dementia-related psychosis. Lastly, while a higher cost burden could be associated with higher morbidity following a psychosis diagnosis as well, analysis of post-diagnosis morbidity is beyond the scope of the current report.

## 6. Conclusions

HCRU and costs for patients with dementia increase in the year after a new diagnosis of psychosis. The increase is observed both in unweighted and weighted mean cost per patient per year analyses. Utilization of acute care was a key driver of increased HCRU and costs immediately following diagnosis, highlighting the importance of appropriate treatment and prompt symptom management in newly diagnosed dementia patients with psychosis. Our data suggest that psychosis is associated with a high cost burden overall for patients with dementia, suggesting an unmet need for timely evaluation and management of dementia-related psychosis, and safe and effective treatments for these patients.

## Figures and Tables

**Table 1:** Annualized all-cause unweighted HCRU claims per patient per year for patients with dementia-related psychosis.

No. of Claims	Sample Size, N	Inpatient	Emergency Department/Observation Stay	Outpatient ^1^	Skilled Nursing Facility	Home Healthcare/Hospice	Physician Visits	Prescription Drug Fills
**All-Cause HCRU**								
Baseline period								
Mean (SD)	49,509	1.0 (1.4)	1.2 (1.9)	5.6 (7.6)	1.2 (2.2)	1.1 (2.1)	26.7 (20.0)	63.0 (43.2)
Median		1.0	1.0	3.0	0.0	0.0	22.0	53.9
1 year post index								
Mean (SD)	49,509	1.7 (5.8)	1.5 (6.0)	6.9 (11.1)	1.9 (4.6)	3.3 (8.6)	38.4 (41.9)	77.4 (55.9)
Median		0.0	0.0	3.0	0.0	0.0	26.5	69.0
2 years post index								
Mean (SD)	26,808	0.9 (2.8)	1.0 (4.3)	5.9 (9.6)	1.0 (2.9)	2.5 (6.0)	28.4 (29.0)	78.3 (55.6)
Median		0.0	0.0	3.0	0.0	0.0	21.0	69.4
3 years post index								
Mean (SD)	16,027	0.8 (2.7)	0.8 (3.9)	5.7 (9.0)	0.9 (3.0)	2.5 (6.1)	27.2 (29.2)	77.8 (57.1)
Median		0.0	0.0	2.8	0.0	0.0	20.0	68.7
4 years post index								
Mean (SD)	9277	0.7 (2.4)	0.8 (2.4)	5.6 (10.3)	0.8 (2.7)	2.5 (6.7)	26.0 (26.5)	77.8 (60.5)
Median		0.0	0.0	2.6	0.0	0.0	19.6	67.6

Data are mean (standard deviation). ^1^ Outpatient setting does not include emergency department or observation hospital stays.

**Table 2:** Annualized psychosis-related unweighted HCRU claims per patient per year for patients with dementia-related psychosis.

No. of Claims	Sample Size, N	Inpatient	Emergency Department/Observation Stay	Outpatient ^1^	Skilled Nursing Facility	Home Healthcare/Hospice	Physician Visits	Prescription Drug Fills
**Psychosis-Related HCRU**								
Baseline period								
Mean (SD)	49,509	0.07 (0.27)	0.04 (0.22)	0.14 (0.42)	0.05 (0.27)	0.02 (0.19)	0.77 (0.95)	2.11 (3.37)
Median		0.00	0.00	0.00	0.00	0.00	1.00	2.00
1 year post index								
Mean (SD)	49,509	0.08 (1.92)	0.03 (0.49)	0.22 (1.54)	0.22 (1.66)	0.12 (1.57)	2.17 (9.19)	7.66 (9.21)
Median		0.00	0.00	0.00	0.00	0.00	0.00	6.00
2 years post index								
Mean (SD)	26,808	0.03 (0.33)	0.01 (0.16)	0.15 (0.92)	0.09 (0.83)	0.05 (0.82)	0.96 (3.69)	6.46 (8.71)
Median		0.00	0.00	0.00	0.00	0.00	0.00	2.55
3 years post index								
Mean (SD)	16,027	0.02 (0.39)	0.01 (0.15)	0.12 (0.85)	0.08 (0.95)	0.05 (1.25)	0.84 (4.35)	5.72 (8.46)
Median		0.00	0.00	0.00	0.00	0.00	0.00	0.00
4 years post index								
Mean (SD)	9277	0.02 (0.26)	0.01 (0.14)	0.12 (0.88)	0.06 (0.68)	0.03 (0.49)	0.71 (3.48)	5.21 (8.56)
Median		0.00	0.00	0.00	0.00	0.00	0.00	0.00

Data are mean (standard deviation). ^1^ Outpatient setting does not include emergency department or observation hospital stays. Abbreviations: HCRU = healthcare resource utilization; SD = standard deviation.

## Data Availability

The data used in this analysis are available for a fee to qualified individuals and institutions from the Centers for Medicare and Medicaid Services, and are subject to the terms of a Data Use Agreement.

## Supplementary Materials

The following are available online at https://www.mdpi.com/article/10.3390/geriatrics7020029/s1, Figure S1: Patient Selection, Table S1: ICD (9th and 10th Revisions) Clinical Modification Codes for Dementia, Table S2: ICD (9th and 10th Revisions) Clinical Modification Codes Used to Define Exclusions for Conditions Potentially Associated with Psychosis, Table S3: ICD (9th and 10th Revisions) Clinical Modification Codes for Manifestations of Psychosis Used to Establish the Presence of Dementia-Related Psychosis, Table S4: Annualized All-cause Weighted HCRU Claims Per Patient Per Year for Patients with Dementia-related Psychosis, Table S5: All-cause Costs Per patient Per Year by Type of Medicare Setting and Overall in Medicare and LTC, Table S6: Psychosis-Related Costs Per Patient Per Year by Setting, Table S7: Annualized Psychosis-related Weighted HCRU Claims Per Patient Per Year for Patients with Dementia-related Psychosis.
