# Supplementary material for: Economic Evaluation of Healthcare Resource Utilization and Costs for Newly Diagnosed Dementia-Related Psychosis
Source: Geriatrics (Basel). 2022 Mar 8;7(2):29. doi: 10.3390/geriatrics7020029 (PMC8938820; doi:10.3390/geriatrics7020029)
Supplement: Supplementary file 1 [file geriatrics-07-00029-s001.zip › geriatrics-1479417-supplementary.pdf]

Healthcare resource utilization and costs for newly diagnosed dementia-related psychosis

Supplement

Table S1. ICD (9th and 10th Revisions) Clinical Modification Codes for Dementia.

Dementia Type	Dementia Diagnosis Codes
Alzheimer's disease and senile dementia	ICD 9: 331.0, 290.0, 290.10, 290.13, 290.21, 797, 331.2<br>ICD 10: G30.0, G30.1, G30.8, G30.9, G31.1
Parkinson's disease dementia	ICD 9: 332<br>ICD 10: G20
Lewy body dementia	ICD 9: 331.82<br>ICD 10: G31.83
Frontotemporal dementia	ICD 9: 331.19, 331.11<br>ICD 10: G31.09, G31.01
Vascular dementia	ICD 9: 290.40, 290.43, 438.0<br>ICD 10: F01.50
Other/unspecified dementia	ICD 9: 294.10, 294.20<br>ICD 10: F03.90, F02.80

Abbreviation: ICD=International Classification of Diseases.

Table S2. ICD (9th and 10th Revisions) Clinical Modification Codes Used to Define Exclusions for Conditions Potentially Associated with Psychosis.

Exclusions	Diagnosis Codes
Chronic psychiatric disease	ICD 9: 295, 296, 297, 298.3, 293.83, 293.84, 301.20, 301.22, 291.1, 291.2, 291.3, 291.5, 292.11, 292.12, 292.82, 293.81, 293.82<br>ICD 10: F20, F21, F24, F25, F30, F31, F32.3, F33.3, V11.0, F1015, F10232, F10250, F10251, F10259, F10950, F10951, F10959, F11122, F11150, F11151, F11159, F11222, F11250, F11251, F11259, F11922, F11950, F11951, F11959, F12122, F12150, F12151, F12159, F12222, F12250, F12251, F12259, F12922, F12950, F12951, F12959, F13150, F13151, F13159, F13232, F13250, F13251, F13259, F13932, F13950, F13951, F13959, F14122, F14150, F14151, F14159, F14222, F14250, F14251, F14259, F14922, F14950, F14951, F14959, F15122, F15150, F15151, F15159, F15222, F15250, F15251, F15259, F15922, F15950, F15951, F15959, F16122, F16150, F16151, F16159, F16183, F16250, F16251, F16259, F16283, F16950, F16951, F16959, F16983, F18150, F18151, F18159, F18250, F18251, F18259, F18950, F18951, F18959, F19122, F19150, F19151, F19159, F19222, F19232, F19250, F19251, F19259, F19922, F19932, F19950, F19951, F19959
Seizures	ICD 9: 345, 310, 333.2, 649.4, 780.33, 300.11, 780.39, 341.81, 333.2<br>ICD 10: F482, G25.3, R56.1, G40, F44.5, R56.9, G93.81, G83.84, G25.3
Chronic alcohol-induced dementia	ICD 9: 291.2<br>ICD 10: F10.27, G312
Drug-induced persisting dementia	ICD 9: 292.82<br>ICD 10: F19.97
Ischemic, hemorrhagic, other stroke	ICD 9: 430, 431, 432.0, .1, .9 433.x1, 434.x1<br>ICD 10: I60.X, I61.X, I62.X, I63.X, I64, I67.X, I69.X except I69.310
Cerebral amyloid angiopathy	ICD 10: I68.0
Central nervous system neoplasm malignancy	ICD 9: 191.X, 192.1, 239.6, 198.3, 225.0, 225.2<br>ICD 10: C70.0, .9, C71.X, C72.9, C721, C7931, C7932, D33.0,.1, 2, .7, .9 D32.0, .9, D49.6
Huntington disease	ICD 10: G10
Vascular malformation	ICD 9: 747.81<br>ICD 10: Q28.2, 28.3
Other	ICD 9: 331.7, 331.81, 331.89, 437.1, 198.3, 192.8, 192.9<br>ICD 10: G13.2, G13.8, G93.7, G31.89, G31.9, G46

Abbreviation: ICD=International Classification of Diseases.

Table S3. ICD (9th and 10th Revisions) Clinical Modification Codes for Manifestations of Psychosis Used to Establish the Presence of Dementia-Related Psychosis.

Disease Manifestation	Psychosis Diagnosis Codes
Psychosis	ICD 9: 293.81, 293.82, 290.8, 290.9, 297.1, 298.0, 298.1, 298.4, 298.8, 298.9, 368.16, 780.1, 290.12, 290.20, 290.42<br>ICD 10: F06.2, F06.0, F22, F23, F28, F29, H53.16, R44.0, R44.1, R44.2, R44.3

Abbreviation: ICD=International Classification of Diseases.

Table S4. Annualized All-cause Weighted HCRU Claims Per Patient Per Year for Patients With Dementia-related Psychosis.

	Sample Size, N	Inpatient	Emergency Department/ Observation Stay	Outpatient[1]	Skilled Nursing Facility	Home Health Care/ Hospice	Physician Visits/DME	Prescription Drug Fills
Baseline period	49,509	1.0	1.2	5.6	1.2	1.1	26.7	63.0
1 year post index	49,509	0.9	1.1	6.5	1.4	2.0	30.8	79.2
2 years post index	26,808	0.6	0.8	5.8	0.9	1.9	26.0	79.3
3 years post index	16,027	0.6	0.7	5.6	0.8	1.9	24.8	78.9
4 years post index	9277	0.5	0.6	5.4	0.7	1.8	23.8	78.5

Weighted mean values are shown. [1] Outpatient setting does not include emergency department or observation hospital stays. Abbreviation: DME=durable medical equipment; LTC=long-term care; SD=standard deviation.

Table S5. All-cause Costs Per patient Per Year by Type of Medicare Setting and Overall in Medicare and LTC.

	Sample Size, N	Inpatient	Outpatient	Skilled Nursing Facility	Home Health Care/ Hospice	Physician Visits/DME	Prescription Drug Fills	Total Medicare	LTC	Total
Baseline period										
Mean (SD)	49,509	9989 (18,376)	2817 (5208)	7721 (15,845)	3279 (7638)	5585 (6615)	4303 (4893)	33,693 (36,360)	15,060 (30,429)	48,753 (46,068)
Adjusted mean		9989	2817	7721	3279	5585	4303	33,693	15,060	48,753
1 year post index										
Mean (SD)	49,509	25,982 (96,608)	3731 (11,872)	11,430 (31,105)	9901 (20,680)	8588 (18,331)	5257 (5992)	64,889 (123,792)	24,495 (36,918)	89,384 (125,116)
Adjusted mean		11,779	3179	8534	6709	5908	5326	41,433	26,270	67,704
2 years post index										
Mean (SD)	26,808	11,971 (49,692)	2775 (6228)	5157 (17,087)	8875 (18,563)	5512 (10,773)	5010 (5751)	39,300 (67,456)	29,136 (40,310)	68,436 (74,816)
Adjusted mean		7357	2590	4584	6698	4605	5142	30,975	30,634	61,609
3 years post index										
Mean (SD)	16,027	10,882 (52,390)	2616 (7040)	4455 (15,603)	8786 (18,710)	5084 (10,103)	4828 (5940)	36,651 (69,457)	32,235 (41,942)	68,885 (77,064)
Adjusted mean		6429	2425	3880	6762	4225	4941	28,662	33,756	62,417
4 years post index										
Mean (SD)	9277	9914 (43,606)	2402 (5153)	3746 (14,124)	8878 (18,992)	4709 (9359)	4653 (6199)	34,302 (60,809)	34,838 (42,976)	69,140 (69,635)
Adjusted mean		6045	2273	3310	6837	3931	4776	27,171	36,645	63,816

All values are 2015 US dollars per patient per year. Abbreviation: DME=durable medical equipment; LTC=long-term care; SD=standard deviation.

Table S6. Psychosis-Related Costs Per Patient Per Year by Setting.

	Sample Size, N	Inpatient	Outpatient	Skilled Nursing Facility	Home Health Care/Hospice	Physician Visits/DME	Prescription Drug Fills	Total
Baseline period								
Mean (SD)	49,509	458 (2603)	57 (270)	150 (1329)	26 (326)	128 (224)	137 (538)	955 (3022)
Weighted mean		458	57	150	26	128	137	955
1 year post index								
Mean (SD)	49,509	2912 (38,279)	156 (1827)	1885 (13,022)	559 (6336)	356 (1803)	795 (1603)	6663 (41,597)
Weighted mean		1147	115	1237	317	227	782	3824
2 years post index								
Mean (SD)	26,808	319 (4814)	66 (689)	392 (5009)	193 (3177)	113 (537)	620 (1480)	1703 (8191)
Weighted mean		256	68	356	137	107	642	1565
3 years post index								
Mean (SD)	16,027	226 (3588)	50 (480)	311 (4005)	166 (2640)	91 (499)	510 (1400)	1354 (6673)
Weighted mean		206	52	292	118	89	523	1280
4 years post index								
Mean (SD)	9277	223 (3458)	54 (511)	202 (2711)	122 (2267)	77 (397)	399 (1284)	1077 (5494)
Weighted mean		170	57	210	116	73	419	1054

All values are 2015 US dollars per patient per year. Abbreviations: DME=durable medical equipment; SD=standard deviation.

Table S7. Annualized Psychosis-related Weighted HCRU Claims Per Patient Per Year for Patients With Dementia-related Psychosis.

	Sample Size, N	Inpatient	Emergency Department/ Observation Stay	Outpatient[1]	Skilled Nursing Facility	Home Health Care/ Hospice	Physician Visits/DME	Prescription Drug Fills
Baseline period	49,509	0.07	0.04	0.14	0.05	0.02	0.77	2.11
1 year post index	49,509	0.05	0.02	0.21	0.17	0.07	1.60	7.64
2 years post index	26,808	0.02	0.01	0.15	0.08	0.04	0.94	6.49
3 years post index	16,027	0.02	0.01	0.12	0.08	0.03	0.82	5.80
4 years post index	9277	0.01	0.01	0.13	0.06	0.03	0.69	5.25

Weighted mean values are shown. [1] Outpatient setting does not include emergency department or observation hospital stays. Abbreviation: DME=durable medical equipment; LTC=long-term care; SD=standard deviation.

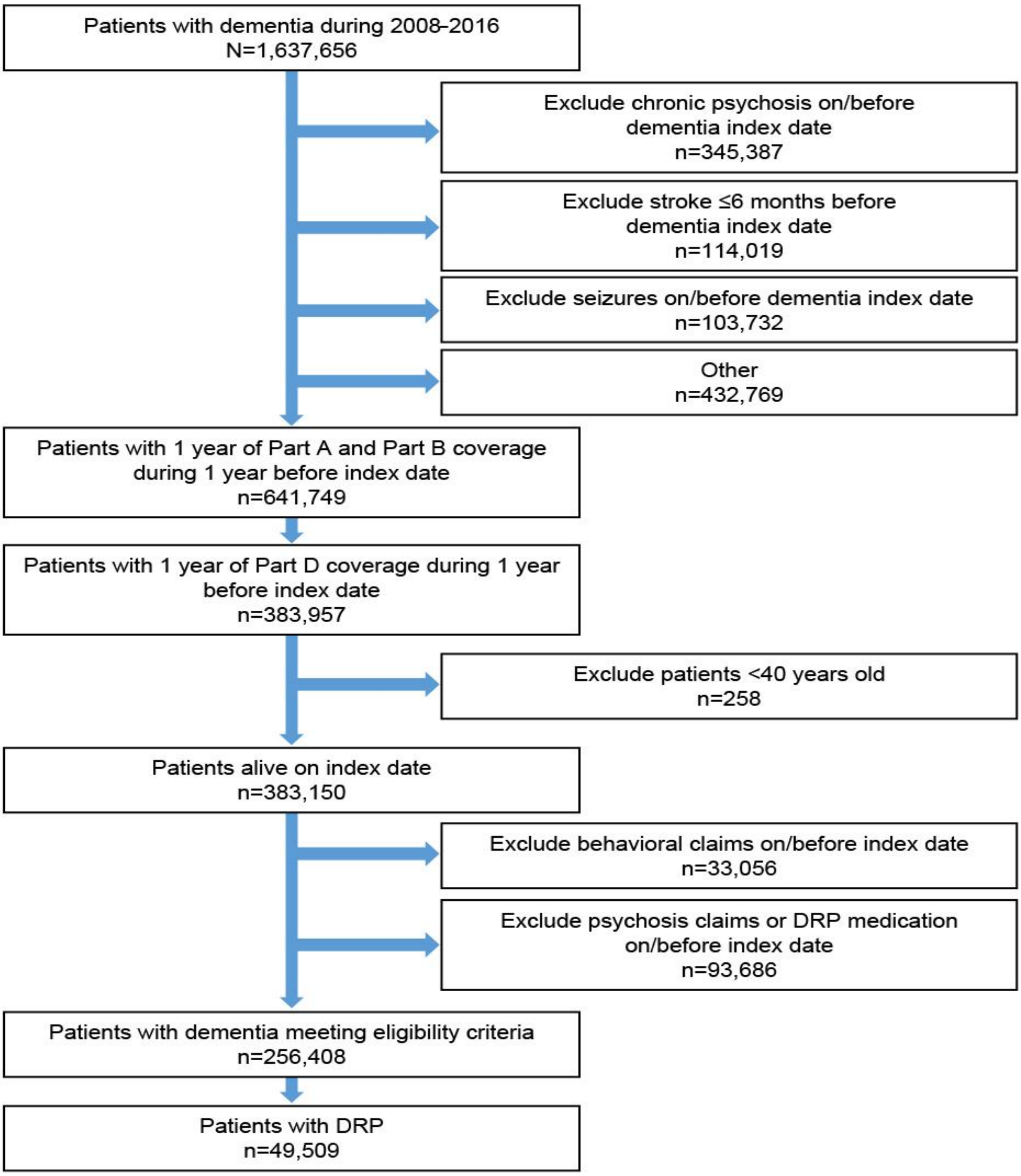

Figure S1. Patient Selection. Abbreviation: DRP=dementia-related psychosis.
